# Pineal Gland from the Cell Culture to Animal Models: A Review

**DOI:** 10.3390/life12071057

**Published:** 2022-07-15

**Authors:** Alekhya Peruri, Alexandra Morgan, Alida D’Souza, Bridget Mellon, Carey W. Hung, Gabriella Kayal, Haejung Shin, Kim Nguyen, Malek Zahed, Mason Yount, Reilly Ellis, Taylor Wynne, Virginia Fritz, Zachary Simmons, Kelly C. S. Roballo

**Affiliations:** 1 Biomedical Affairs and Research, Edward Via College of Osteopathic Medicine, 2265 Kraft Drive, Blacksburg, VA 24060, USA; aperuri@vt.vcom.edu (A.P.); agreen01@vt.vcom.edu (A.M.); adsouza@vt.vcom.edu (A.D.); bmellon@vt.vcom.edu (B.M.); chung@vt.vcom.edu (C.W.H.); gkayal@vt.vcom.edu (G.K.); hshin@vt.vcom.edu (H.S.); knguyen01@vt.vcom.edu (K.N.); mzahed@vt.vcom.edu (M.Z.); myount@vt.vcom.edu (M.Y.); rellis01@vt.vcom.edu (R.E.); twynne@vt.vcom.edu (T.W.); vfritz@vt.vcom.edu (V.F.); zsimmons@vt.vcom.edu (Z.S.); 2 Department of Biomedical Sciences and Pathobiology, Virginia Maryland College of Veterinary Medicine, Virginia Tech, 1410 Prices Fork, Blacksburg, VA 24060, USA

**Keywords:** melatonin, regeneration, neurodegenerative process, cell culture, animal models

## Abstract

This review demonstrates current literature on pineal gland physiology, pathology, and animal model experiments to concisely explore future needs in research development with respect to pineal gland function and neuro-regenerative properties. The pineal gland plays an integral role in sleep and recovery by promoting physiologic circadian rhythms via production and release of melatonin. Yet, the current literature shows that the pineal gland has neuroprotective effects that modulate both peripheral and central nerve injuries through several direct and indirect mechanisms, such as angiogenesis and induction of growth factors and anti-inflammatory mediators. Animal models have also shown correlations between pineal gland function and metabolic homeostasis. Studies have shown that a functional pineal gland is essential in preventing and slowing the progression of certain diseases such as diabetes, osteoporosis, vertebral osteoarthritis, and neurodegenerative processes. Lastly, the array of cell culturing methods and animal models that can be used to further develop the study of pineal gland function and nervous system injury were reviewed.

## 1. Major Functions of Pineal Gland

### 1.1. Pineal Gland Overall Morphology, Anatomy, and Function

**Figure 1:**
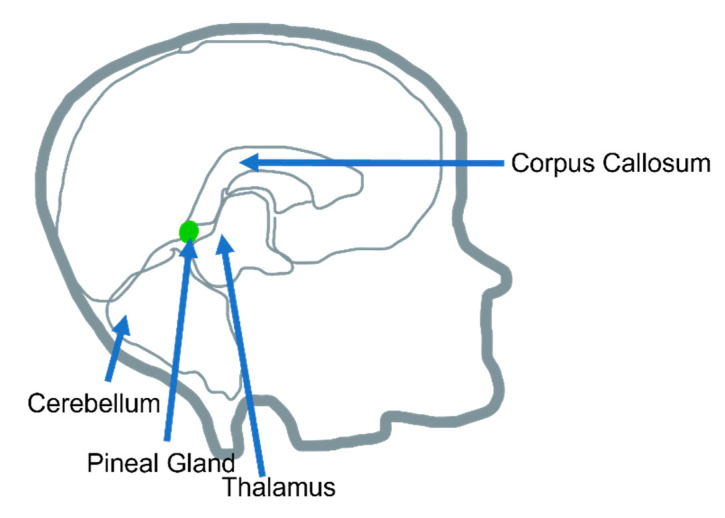
Pineal gland anatomic location.

The endocrine system, made up of the body’s various hormones, regulates several biological processes in the human body. Of the endocrine organs, the pineal gland is highlighted in this review article. Located in the posterior aspect of the cranial fossa, as part of the epithalamus, the pineal gland encounters the third ventricle (Figure 1), which is outside the blood–brain barrier [1]. In mammals, the main cell types in the pineal gland are pinealocytes (95%), followed by scattered glial cells (astrocytic and phagocytic subtypes) [2].

The primary function of the pineal gland is to produce and secrete *N*-acetyl-5methoxy-tryptamine, also known as melatonin [3]. The pinealocytes are responsible for the synthesis and secretion of the hormone melatonin, which controls circadian cycles [4]. This hormone is a nocturnal one and is rhythmically produced in response to changes in light [5], with the highest blood levels peaking in the middle of the night [5]. Once produced, it is not stored but instead released into circulation to carry out its actions both centrally and peripherally [5].

Melatonin binds to G-protein-coupled receptors (MT1 and MT2) in the hypothalamus, pars tuberalis, and suprachiasmatic nucleus of the brain (among other structures) as well as receptors in the immune system, kidneys, and vessels [5]. The MT1 receptor appears to have an increased affinity for melatonin, and binding leads to inhibition of neuron firing at the suprachiasmatic nucleus, the regulator structure of circadian rhythm in the anterior hypothalamus [4,5,6,7,8,9].

Regarding its many functions mentioned, a prominent function of melatonin is the establishment of circadian rhythm and induction of sleep. Melatonin signaling begins in the photoreceptor cells of the retina. Light-stimulated ganglion cells transmit their signal through the retinohypothalamic tract to the suprachiasmatic nucleus of the hypothalamus. From there, the signal takes a circuitous pathway through the hypothalamic paraventricular nuclei, thoracic cord, and sympathetic ganglia to arrive at the pineal gland, at which time norepinephrine exerts a suppressive effect on the melatonin pathway [6].

Melatonin is also known to exert a neuroprotective effect, stimulating activity of glutathione peroxidase, an antioxidant enzyme, in the brain [5]. Glutathione peroxidase reduces H_2_O_2_, which accumulates under conditions of cellular stress, to less-reactive compounds. Melatonin inoculation in mice has been shown to upregulate this enzyme in all tissues, not just the brain [10]. Mechanistically, there is evidence to suggest that melatonin reduces innate immune cell migration and increases mitochondrial oxidative phosphorylation processes [11]. This has potentially significant clinical implications in treatments for Alzheimer’s disease, reduction of infarction damage in stroke, as well as neuroprotection in hypoxic neonates [11].

It is known that melatonin production begins with tryptophan [1]. Conversion through a multi-step pathway leads to the production of serotonin, which eventually travels to the pineal gland [1]. Here, it is converted to melatonin in a cyclic, light-dependent process as described above. As a result of the pineal gland’s location, tumors or cyst formations are not uncommon. Compression of this structure leads to pressure on the aqueduct of Sylvius, the structure allowing the cerebrospinal fluid (CSF) to circulate out. Sequential hydrocephalus due to build up in fluid can lead to a clinical presentation with nausea, vomiting, visual changes, seizures, and memory alterations [1,12,13,14].

### 1.2. Neuronal Interactions with the Hypothalamus

The pineal gland functions to synthesize and release melatonin in a circadian pattern, with peak secretion occurring at nighttime. Regulation of this secretion occurs through afferent signals from the suprachiasmatic nucleus of the hypothalamus, to stimulate the rate-limiting enzyme in melatonin synthesis, arylalkylamine *N*-acetyltrasnferase (AANAT), thereby functioning as the nervous systems internal clock [13]. The release of melatonin, as controlled by the hypothalamus, following the rhythmic cycle between day and night, allows melatonin to exert its physiologic effects during the nightly period [13].

The production and secretion of melatonin from the pineal gland peaks in periods of darkness following the circadian oscillation of day and night cycles. The pineal gland in humans has evolutionarily lost the direct ability to sense light [14]. Yet, humans developed a photopigment in the retinal ganglion cells, melanopsin, which can regulate the signaling pathway required for the release of melatonin. In periods of prolonged darkness, the suprachiasmatic nucleus receives signals from melanopsin, thereby stimulating the release of melatonin [14]. The pineal gland can either secrete melatonin into the blood or the CSF. The melatonin within the CSF is more regulated than in the blood, indicating that melatonin’s act in maintaining the body’s circadian rhythm is due to its presence in the CSF rather than peripherally [15].

Prostaglandins are released by the pineal gland and the median eminence of the hypothalamus under stimulation by norepinephrine, acting through alpha adrenergic receptors and the increased activity of intracellular adenylate cyclase [16]. Prostaglandins then aid in the release of melatonin from the pineal gland and GnRH form the median eminence through receptors found on their respective membranes [16].

### 1.3. Pineal Gland and Its Correlation with Illness

Within the field of pineal gland research, physiologic functions and regeneration of the pineal gland has been extensively and continuously researched. Structures within the mammalian central nervous system fail to regenerate as effectively as peripheral nerves [17]. One study posed that the glial cell environment surrounding nerves determines successful regeneration [18]. Others found significant differences in growth factors and waste-clearance rates between the central nervous systems a peripheral nervous system, which may allow peripheral nerves to be more efficient at healing [17,19].

**Figure 2:**
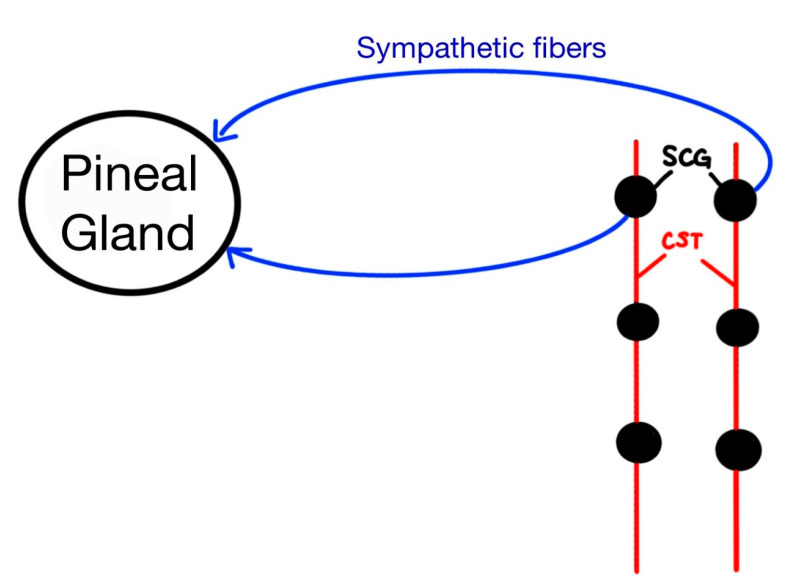
A diagram of the pineal gland innervations to the superior cervical ganglion (SCG) and the cervical sympathetic trunk (CST).

The pineal gland is linked with sympathetic fibers from the cervical sympathetic trunk (CST), which impacts the enzyme serotonin *N*-acetyltransferase (NAT) (Figure 2). NAT plays a large role in maintaining circadian rhythm. One study examined the effects of pineal gland function after regeneration of sympathetic cervical ganglions (SCG) [20]. Within this study, the CSTs were cut after 36 h, and levels of NAT were then examined. Three months later, NAT levels only increased 14% compared to controls. They found that neither the density of preganglionic fibers nor the gland’s ability to stimulate NAT were affected. Additionally, the number of CSTs, action potential velocity, and the number of synapses were unchanged. The authors did find decreased mean diameters of the preganglionic CST axons and choline acetyltransferase levels. Additionally, electrical stimulation was found to be the least effective in increasing NAT levels [20]. Overall, the study showed that preganglionic fibers synapse with a specific postsynaptic receptor under normal circumstances. After ligation of the CST fibers, the regenerating preganglionic innervations “incorrectly” synapse with different postganglionic fibers, which resulted in depressed NAT levels [20]. More research could be done to study different enzyme concentrations, receptors, and functions within the pineal gland in order to further explore its function and to determine whether common markers exist amongst all preganglionic fibers (Figure 2).

Melatonin may also play a role in the inflammation [21,22,23,24,25,26]. Inflammation is part of the illness process, which is modulated by diverse compounds release by cells such as cytokines and interleukins. As soon as the inflammation starts, anti-inflammatory response also begins, which is also modulated by other cytokines, interleukins, and some hormones such as cortisol and melatonin. Melatonin, the pineal gland’s major product, exerts anti-inflammatory, immunomodulatory, and anti-cancer effects on the human body. Melatonin production decreases with age, which suggests it may prove to be a useful target in the analysis of many age-related processes and disorders [24,25,26].

In a recent study, telomere dysfunction was associated with decreased melatonin production in elderly mice [24]. Telomeres function to promote cellular stability and replication and are, therefore, a widely accepted marker for endothelial cell dysfunction in the setting of atherosclerosis. Most notably, melatonin treatment reduced intimal thickening and endothelial apoptosis in the mouse model, further supporting melatonin’s beneficial cardiovascular properties [24].

Furthermore, melatonin has also been shown to have beneficial vascular effects in the setting of COVID-19 [25]. Melatonin significantly reduced the frequency at which COVID-19 patients experienced thrombotic events, septicemia, and even death when given in addition to the standard treatment protocol for hospitalized patients. Many hospitalized COVID-19 patients have comorbidities such as hypertension, hyperlipidemia, and diabetes mellitus. Melatonin may be acting on these disease processes to reduce COVID-19 mortality. Overall, the use of melatonin has protective properties in the setting of COVID-19 inpatient treatment [25].

Melatonin has been hypothesized to have anti-cancer effects. The hormone was found to dramatically reduce levels of vascular endothelial growth factor (VEGF) and reactive oxygen species in a hypoxic environment [26]. VEGF is an essential component for cancer angiogenesis and is a current target of many immunotherapies. Melatonin has also been shown to upregulate apoptosis of malignant cells and downregulate their production, specifically in the setting of colorectal cancer [27]. The method by which melatonin exerts its apoptotic effects remains incompletely understood, but Bcl-2 and Notch1 are two of the suspected targets involved. These findings suggest melatonin is capable of immunomodulation and may serve as a potential therapeutic target for future cancer treatment [27].

In another recent study, it was pointed out that adolescents with impaired insulin metabolism had markedly lower levels of nocturnal melatonin relative to their healthy counterparts. This finding suggests melatonin may have significant action on metabolic processes as well as circadian rhythms [28].

### 1.4. Pineal Gland and It’s Relationship with the Central and Peripheral Nervous System Illness

While the effects of the central nervous system on the pineal gland is significant, researchers continue to investigate the effects of the peripheral nervous system’s influence on the pineal gland. Pazo and Gonzalez (1991) [29] compared the action potential responses of pinealocytes to afferent neurotransmission signaling from retinal stimulation through light activation and electrode stimulation of the suprachiasmatic nucleus in the central nervous system, the SCG of the autonomic nervous system, and the sciatic nerve of the peripheral nervous system. Of the pinealocytes that responded to both central and peripheral nervous system afferent signals, all were completely inhibited by the sciatic nerve, and only three-fourths were inhibited by the SCN, SCG, and light stimulation [29]. This finding is important because it suggests that the peripheral nervous system and central nervous system influence underlying biological mechanisms of the pineal gland besides light triggering the circadian rhythm in the central nervous system.

The pineal gland’s secretion of melatonin, a hormone most known for its release and participation in the circadian sleep–wake cycle of mammals, has also been found to reduce free radical accumulation and enhance neuroprotective mechanisms of neurons, especially when injured [30,31]. In an experiment testing the role of melatonin in driving angiogenesis within ischemic nervous tissue of spinal cord injuries, Yingli et al. (2017) [31] found a significant increase in endothelial cell biomarkers (i.e., CD31) in rats with spinal cord injuries that were receiving melatonin treatment compared to rats with spinal cord injuries not receiving exogenous melatonin treatment. Furthermore, fluorescent markers added to the tissue samples to evaluate the degree of reperfusion within these blood vessels showed a significant decrease in blood vessel perfusion of spinal-cord-injured rats without melatonin treatment compared to the sham rats, which was expected; however, melatonin administration to spinal-cord-injured rats showed a significant increase in perfusion of the injured blood vessels [31]. These results suggest that melatonin plays a role in modifying damaged blood vessels to promote the reperfusion of tissue, which leads to less ischemia and possibly a rescue of its neuronal function. In this study, it was concluded that melatonin treatment can ameliorate neuronal stress by inducing the rescue of blood vessel formation and reperfusion [31]. This conclusion is valuable in terms of further investigation of neuronal repair since damage to the spinal cord often leads to corresponding damage of peripheral nerves that extend from the central nervous system, such as motor axons.

**Figure 3:**
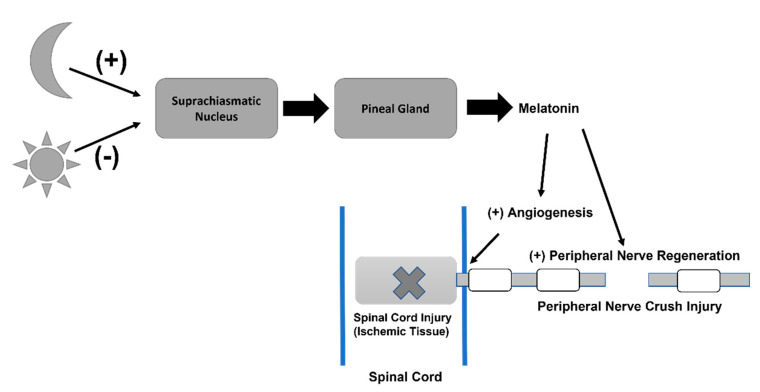
Overall Pineal Gland action into the Central and Peripheral Nervous injury. In response to the external and internal stimuli, pineal gland and the peripheral nervous system work together to regulate the body’s needs for repair to restore proper functioning.

Stazi et al. (2021) [30] sought to investigate melatonin effects on restoring presynaptic neuromuscular junction (NMJ) function of motor axons exposed to neurotoxin Alpha-LTx and evaluate the restoration of growth of crushed sciatic motor nerves. Results showed a significant increase in the quantity of NMJ terminals present with functional presynaptic activity in the melatonin-treated motor neurons compared to the non-melatonin-treated group of neurons after subjection to the neurotoxin. Furthermore, exposure of the crushed nerves to melatonin treatment 18 or 26 days after being severed relayed an increase in recovery of neurotransmission upon stimulation, and elongation of the axons was visualized 12 days after injury through immunostaining techniques using a green fluorescent protein marker to track growth [30]. This suggests that melatonin plays a role in promoting the regeneration of damaged peripheral nerve axons. Thus, the pineal gland and the peripheral nervous system work together to regulate the body’s needs for repair to restore proper functioning (Figure 3).

## 2. In Vitro Models of Culturing Pineal Gland Cells

### 2.1. Two-Dimensional (2D) In Vitro Cell Culture

The 2D cell culture of pineal gland cells serve as an important in vitro experimental technique to gain an understanding of the functional and secretory aspects of pinealocytes. Two-dimensional in vitro cell culture also provides researchers with the unique ability to investigate the potency and differentiation of embryonic pineal gland cells to better understand the formation and function of the pineal gland [32]. While monolayer cell culture models, such as 2D culture, are limited in their ability to demonstrate the cell-to-cell communication and coordination of pinealocytes and the glial cells (supporting cells) within the gland, this in vitro model can still provide great insight into the complex endocrine and neurologic functions of pinealocytes [33].

The procedure for pineal gland cell culture has been standardized since it was first described in the 1970s [34]. If an isolated pinealocyte culture is desired, the enzyme papain is required to digest and extract the pinealocytes. The process consists of removing the rat’s pineal gland or pineal gland from the chosen model animal species shortly after decapitation and immediately placing the pineal gland in ice-cold medium (Dulbecco’s Modified Eagle’s Medium (DMEM) with supplements). This is followed by enzymatic digestion by papain, and the cells are then resuspended in a medium of DMEM with 10% fetal calf serum and antibiotics. Finally, cells in the medium are incubated 37 °C in 5% CO_2_/95% air for 24 h. Following the 24 h incubation, the culture medium and pinealocyte suspension will be removed from the flask (the astrocytes will remain attached to the flask and can also be cultured separately if desired) and centrifuged and resuspended in DMEM to a final concentration of 2 × 10^5^ cells/mL [35]. Another method to isolate and culture pinealocytes is to extract the pineal gland from 2-day-old rats, wash the pineal gland with buffer solution, and resuspend the cells with 0.25% trypsin for 5–10 min with slow agitation, followed by filtration and centrifugation (130× *g* for 10 min). Pinealocytes then can be seeded in a 12-well plate with cell culture media (DMEM with 20% FBS calcium–magnesium-free) and incubated [36]. Once created, these 2D pinealocyte cultures can be co-cultured with other astrocytes and/or neurons to study their interactions in vitro and functional and secretory properties. For melatonin quantification in cell culture, this can be achieved by isotope dilution mass spectrometry [37].

### 2.2. Three-Dimensional (3D) In Vitro Cell Culture

Cell culture is a common technique used to study the effect of drugs and toxic compounds on cells as well as carcinogenesis and stem cell study. While in vitro cell cultures are mostly cultured using 2D models, there has been an increasing number of studies exploring 3D cell culturing techniques in the past years as a way to be more translational to the in vivo model. Although a relatively novel technique, 3D cell culture has quickly gained popularity in many fields of science, including virology, cardiovascular medicine, and oncology. Lv et al. (2017) [38] explained that 3D cell culture is a way to bridge the gap between conventional 2D in vitro and animal testing models because the 3D cell culture microenvironment considers not only the cell but also the spatial organization and extracellular matrix (ECM) of the cell. This study describes seven methods of 3D tumor cell culture, including static suspension culture, hanging drop, magnetic levitation, spinner/rotational based approaches, microfluidic device, gel embedding, and scaffolds. After several analyses, it was concluded that gel embedding and scaffolds are the best methods to resemble in vivo conditions by being able to provide a rich and controlled ECM network with desired growth factors. This research provided a comprehensive resource for others to use since it describes the advantages and disadvantage of each of the seven 3D cell culture methods.

As a novel technique, 3D cell culture is a complex technique that often requires specialized equipment and expensive resources. Maritan et al. (2017) [39] described a rapid and flexible protocol for aggregating cells into multicellular 3D spheroids of consistent size that is compatible with a variety of cell lines. The protocol aims to produce spheroids (3D cell organization) by cell aggregation, which includes creating a methyl cellulose solution and spheroid formation medium and producing the cell spheroid. The authors also included three ways to quantify invasion, including brightfield microscopy, fluorescence microscopy with live cell stain, and immunofluorescence. Each step of this protocol is described in a succinct and clear manner allowing for easy reproducibility. Using the detailed protocol described in this research will allow many scientists to generate and utilize 3D cell spheroids to replicate the 3D microenvironment of tissues and model in vivo growth of desired cells, such as pineal gland cells. Moreover, the robust application of this protocol may lead the way in creating the ideal microenvironment for pineal cell aggregates [39].

### 2.3. Alternative Culture Methods

In vitro “organ” culture can be an alternative method for cultivating pinealocytes. According to Clinton, J. and McWilliams-Koeppen, P. (2019) [40], it involves the use of organoids hat are artificially grown micro-tissues derived from patients or animal models. This study describes organ cultures as more advantageous than conventional 2D cell culture because organoids can self-organize into complex, functional tissue structures that represent the diverse cellular components and characteristic morphology of in vivo organs [40].

The method for organ culture is not strictly confined to the specific procedural steps listed above. For instance, a study performed by Khavinson et al. (2011) [41] revealed that the addition of the peptide Ala-Glu-Asp-Gly to organ culture stimulated the proliferation and secretory activities of rat pinealocytes. The study suggests that this method can be used to improve recovery and reduce the aging process of pineal glands.

Another alternative method includes suspension culture using neonatal rat pineal glands. Pineal glands removed from 2-day-old rats can be prepared with DMEM, fetal calf serum (FCS), and bovine serum albumin (BSA). This medium is replaced with calcium- and magnesium-free DMEM with trypsin. Cells are collected after several steps, which include incubation, centrifugation, removal of supernatant, rinsing, resuspension, and filtration. Trypan blue exclusion revealed greater than 90% viable cells. Microscopy confirmed preserved morphology. Increased enzyme activity was observed for the cultures treated with l-norepinephrine (*N*-acetyltransferase). The biochemical similarities between this dispersion method and organ culture or in vivo pineal glands suggests that this method can be helpful in biochemical and morphological research on the pineal gland [36]

## 3. In Vivo Animal Model for the Study of Pineal Gland

### 3.1. Non-Genetically Modified Animal Models

#### 3.1.1. Swine

Swine are often used for biomedical research because of the similarities between the structure of a pig’s brain and a human’s brain. Swine have been used as a model for cystic fibrosis and Parkinson’s disease [42]. One study that demonstrated the orexinergic central innervation of the pineal gland of the pig was able to analyze the locations and orientations of each structure and the nerve fiber numbers and distribution and compare that to humans and other common animal models [43]. While this research does not specifically discuss swine models in relation to pineal gland disorders and tissue regeneration, it can provide evidence that swine models will be a better match in terms of translating from animal models to humans.

#### 3.1.2. Rabbit

In a study performed in rabbits, Aguilar-Roblero and González-Mariscal (2020) [44] discussed the factors that can impact a rabbit’s circadian rhythms. In terms of the pineal gland, they found that the cervical sympathetic system causes an increase in melatonin release more so than the perception of light [44]. They also analyzed the release of melatonin in relation to rabbits being nocturnal animals. Lastly, they advocated for using rabbit models more often in biomedical research, specifically for glaucoma and heart disease [44].

Another study performed in rabbits investigated the idea that melatonin can be protective and help reduce intervertebral disc degeneration. After inducing intervertebral disc degeneration in rabbit models, one group was injected with melatonin, another with melatonin and a ERK inhibitor, and the last group was injected with saline [45]. They found that the melatonin group had more chondrocytes and collagen II but decreased collagen X compared to the other two groups. Their evidence supports the idea that melatonin can be used to reduce intervertebral disc degeneration by decreasing nucleus pulposus cell degeneration and apoptosis and increasing collagen II formation [45].

#### 3.1.3. Rodents

In a study performed in rats, researchers took a sample size of 28 adult male Sprague–Dawley rats to examine the effects of topical and systemic melatonin administration on wound healing in rats that underwent pinealectomy [46]. They had found that the rats who underwent pinealectomy had delayed wound healing [46]. Of those rats who also received systemic melatonin treatment, there was an increased amount of collagen noted in wound tissue, especially when compared to the topical melatonin-administration group [46]. However, wound surface area was maintained at control level in the group that received topical melatonin treatment [46]. The evidence found in their study suggests that the effect of melatonin on collagen may be controlled by systemic regulation [46].

Another study examined the effects of pinealectomy and melatonin treatment on neuroma formation in transected sciatic nerves in rats [47]. An increase in connective tissue typically led to neuroma formation [47]. In the rats who had undergone pinealectomy without melatonin treatment, there was a significant increase in connective tissue despite a significant decline in thickness of myelin sheath and axon reduction [47]. The rats that had undergone pinealectomy with melatonin treatment had a greater axon count compared to the rats that had undergone pinealectomy without melatonin treatment [46]. These results may suggest that melatonin inhibits collagen synthesis and fibroblast cell proliferation as it relates to neuroma formation [47].

In a study performed with Wistar rats, the researchers denervated, cultured, and stimulated the pineal gland with norepinephrine to initiate melatonin synthesis [48]. Researchers concluded that pineal melatonin production is temporarily inhibited by TNF-α, which is also responsible for neutrophil migration to the site of injury [48]. Nocturnal production in melatonin is impaired initially during an inflammatory response, but it is reestablished shortly afterwards. The researchers concluded that the transcription of AA-NAT, which is an enzyme involved in melatonin synthesis, is modulated by the anti-inflammatory effects of corticosterone and the pro-inflammatory effects of TNF-α [48].

Besides the circadian control, melatonin has also other properties. A recent study showed the importance of melatonin in traumatic brain injury. In this study, a heme oxygenase-1 (HO-1) cAMP response element-binding protein (CREB) rat model was used, and the authors found that melatonin mitigates traumatic-brain-injury-induced depression via HO-1/CREB pathway, thus showing melatonin’s neuroprotective properties in traumatic brain injury [49].

Furthermore, studies using animal model to investigate Alzheimer’s disease (AD), which is a progressive irreversible neurodegenerative disorder, have shown that melatonin has also neuroprotective properties in AD, demonstrated in vivo and in vivo in preclinical studies [50], leading to the reduction of the AD progression in the patient condition.

Another animal model study performed in mice investigated novel drug-based therapies that could be used to reduce the onset of status epilepticus (SE), and the authors hypothesized that melatonin is a therapeutic target for neurodegenerative diseases such as AD, Parkinson’s disease, and epilepsy. To test their hypothesis, the authors used ICR mice, inducting to SE with kainate (KA); after a series of tests, the authors identified a compound indole derivate 3e containing 2-furyl fragment, which is effective against KA-induced SE and oxidative effects and is similar to melatonin [51]. The extent of the therapeutic potential of melatonin is still unclear, but part of the melatonin neuroprotective effect has been explained by the presence of melatonin receptors in special melatonin receptor 1 in the injured cells [52].

### 3.2. Genetically Modified Animal Models for Pineal Gland Study

#### Common Animal Models Used for Specific Pineal Gland Diseases and/or Research

To investigate how disease states affect the pineal gland, some studies used gene editing in animal models. One study used knockout mice with an eye and pineal gland *Rax* gene deletion [53]. The researchers specified that the mutation had to be applied only to the eye and pineal gland, as an unconditioned mutation that would lead to a forebrain malformation [53]. The knockout mice were not able to breed, so the researchers had to generate the mice, and the offspring pattern followed a Mendelian ratio [53]. The following outcome may not be consistent with a more complicated animal model. The researchers had to pick a model where mating, breeding, and genetic predilection were simple enough to oversee, yet the development of the animal could be compared to a human model. Ultimately, the authors concluded that the *Rax* gene contributes to pineal gland expression due to the absence of retinal structures, suprachiasmatic nucleus (SCN), and lack of bodily temperature regulation and circadian rhythm [36]. The authors provided insight on how norepinephrine could be used to mimic the effects of the SCN, so rhythmic gene expression can be studied [53]. It would be beneficial if there was more research on how the rodent model may be different from a human or other mammalian model. Rodents and humans have similar brain structure, but it is unclear how the differences in brain development could affect the role of the *Rax* gene applied to a human model versus the rodent model.

Lewczuk, Prusik et al. (2018) [54] focused on how diabetes affects the pineal gland. The researchers used a pig model for the study because the physiological response in pigs is similar to that in humans with respect to metabolic disorders [54]. In the study, pigs were given streptozotocin to induce diabetes [54]. Six weeks later, they were euthanized, and the pineal gland was removed and studied [54]. The results showed that diabetes affected the sympathetic neurotransmission in the pineal gland, which induced changes in pinealocytes, and diabetes led to a decrease in serotonin levels [54]. Although the pig model is similar to humans, it is certainly not identical. Humans experience extrinsic stimuli independent of diabetes that can result in additional psychological and physical stress that may impact the function of the pineal gland.

Another study explored how a pinealectomy and the removal of melatonin in a sheep model affects bone mineral density (BMD) [55]. Past BMD studies used rat models, but these researchers chose to use a sheep model in attempt to better resemble human physiology [55]. The authors explained that in sheep, an ovariectomy results in a large decrease in BMD like in humans, and rat bone remodeling is different from humans [55]. However, compared to humans, sheep bone loss is lower, so their condition favors osteopenia rather than osteoporosis [55]. Despite that, the results showed that the BMD in the sheep with a pinealectomy was lower, and bone resorption was higher, which demonstrated that the pineal gland played a role in bone remodeling in larger animal models [55].

In many of the studies regarding pineal gland research, a disease is induced in the animal models because these processes are not always naturally occurring. It would be interesting to examine how closely these induced states resemble the naturally occurring pathophysiological processes in a human. Many diseases have genetic and environmental components, so it may be of benefit to see if anything could be adjusted in the induction of diseased states to make them a more accurate representation of human disease.

### 3.3. The Ideal Animal Model for Pineal Gland Study

Unfortunately, even with many anatomical and physiological similarities, humans are vastly different as a structural unit in comparation to our primary animal models, such as mice. Many articles emphasize similarities in human and mouse DNA, but those genes do not always have the same end function when compared. For this reason, the potentially groundbreaking medical advances found in mice likely never proceed from that point to human clinical trials. If they do, the trials are often ineffective or harmful. This is the primary indication and driving force for a new candidate in general research and, more specifically, pineal gland research.

What other animal model can be used going forward? The answer to that is difficult, as there are more than 6000 mammalian species on planet Earth, so this must be narrowed down [56]. Exclusion criteria include non-mammalian animals, mammals with low reproductive rates, and mammals commonly used in research today, such as mice, rats, rabbits, sheep, and pigs. After extensive literature review, there are three intriguing potential candidates: chinchillas, guinea pigs, and the nonhuman primates (NHPs). The most beneficial of the three mentioned is the NHP and, more specifically, the macaque monkey.

To correlate the reasoning for NHP use in research regarding the pineal gland and its potential, it is important to refer to H. Kunzle (1977), who first discovered the relationship between the basal ganglia, the somatosensory cortex, and the thalamus [57]. The basis of his breakthroughs has been further developed and is being used every day in the 21st century to treat Parkinson’s disease. The potential for the macaque to help the scientific community in pineal gland research and more is of similar significance to H. Kunzle’s. However, the NHP animal model can only be used when either no other research model can give significant results or when the researcher is nearing human clinical trials during the translational stage. Due to their similarity in brain mass, brain structure, and 98% DNA similarity, the NHP will be of most benefit in future research regarding peripheral nerve injury, pineal gland function, and neuroscience research overall.

## 4. Pineal-Gland-Derived Compounds Other Than Melatonin

The principal hormone produced by the pineal gland is the melatonin [1]. However, as part of the melatonin synthesis, tryptophan and serotonin are also biproducts of the pinealocytes [1]. Other studies have shown that aromatic-L-amino acid decarboxylase (AADC) and indolethylamine-*N*-methyltransferase (INMT), which are part of the biosynthesis of *N*,*N*-dimethyltryptamine (DMT), were also present in pinealocytes [58]. Arylalkylamine *N*-acetyltransferase (AANAT) and/or *N*-acetyl-serotonin methyltransferase (ASMT) (formerly hydroxyindoleO-methyltransferase (HIOMT)) have also been identified in this gland [49]. Thus, it is possible to affirm that the pineal gland is a source of important biological compounds besides melatonin that also influence major functions in the brain.

## 5. Future Directions

The pineal gland is part of the endocrine and nervous systems. It is located in the middle of the brain and connected to the floor of the third ventricle outside the blood–brain barrier. In mammals, the main cell types in the pineal gland are pinealocytes (95%), followed by scattered glial cells (astrocytic and phagocytic subtypes). Pinealocytes are responsible for the synthesis and secretion of the hormone melatonin, which controls circadian cycles. Low levels of melatonin production have been related to the development of neurodegenerative disorders, thereby indicating a neuroprotective function for this hormone in the central nervous system. However, it is unclear whether the pineal gland could also have a neuroprotective function in peripheral nerves. It is essential to understand more about the function and abilities of the pineal gland, including hormones that may be associated physiologically with the tissue regeneration process.
